# Prenatal *BRCA1* epimutations contribute significantly to triple-negative breast cancer development

**DOI:** 10.1186/s13073-023-01262-8

**Published:** 2023-12-06

**Authors:** Oleksii Nikolaienko, Hans P. Eikesdal, Elisabet Ognedal, Bjørnar Gilje, Steinar Lundgren, Egil S. Blix, Helge Espelid, Jürgen Geisler, Stephanie Geisler, Emiel A. M. Janssen, Synnøve Yndestad, Laura Minsaas, Beryl Leirvaag, Reidun Lillestøl, Stian Knappskog, Per E. Lønning

**Affiliations:** 1 K.G. Jebsen Center for Genome-Directed Cancer Therapy, Department of Clinical Science, University of Bergen, Bergen, Norway; 2 Department of Oncology, Haukeland University Hospital, Jonas Lies Vei 65, N5021 Bergen, Norway; 3 Department of Hematology and Oncology, Stavanger University Hospital, Stavanger, Norway; 4 Cancer Clinic, St. Olavs Hospital, Trondheim University Hospital, Trondheim, Norway; 5 Department of Clinical and Molecular Medicine, Norwegian University of Science and Technology, Trondheim, Norway; 6 Department of Oncology, University Hospital of North Norway, Tromsø, Norway; 7 Department of Surgery, Haugesund Hospital, Haugesund, Norway; 8 Department of Oncology, Akershus University Hospital, Lørenskog, Norway; 9 Institute of Clinical Medicine, University of Oslo, Oslo, Norway; 10 Department of Pathology, Stavanger University Hospital, Stavanger, Norway; 11 Department of Chemistry, Bioscience and Environmental Engineering, Stavanger University, Stavanger, Norway

**Keywords:** Triple-negative breast cancer, *BRCA1*, Methylation, Epimutation, Constitutional epimutation

## Abstract

**Background:**

Normal cell *BRCA1* epimutations have been associated with increased risk of triple-negative breast cancer (TNBC). However, the fraction of TNBCs that may have *BRCA1* epimutations as their underlying cause is unknown. Neither are the time of occurrence and the potential inheritance patterns of *BRCA1* epimutations established.

**Methods:**

To address these questions, we analyzed *BRCA1* methylation status in breast cancer tissue and matched white blood cells (WBC) from 408 patients with 411 primary breast cancers, including 66 TNBCs, applying a highly sensitive sequencing assay, allowing allele-resolved methylation assessment. Furthermore, to assess the time of origin and the characteristics of normal cell *BRCA1* methylation, we analyzed umbilical cord blood of 1260 newborn girls and 200 newborn boys. Finally, we assessed *BRCA1* methylation status among 575 mothers and 531 fathers of girls with (*n* = 102) and without (*n* = 473) *BRCA1* methylation.

**Results:**

We found concordant tumor and mosaic WBC *BRCA1* epimutations in 10 out of 66 patients with TNBC and in four out of six patients with estrogen receptor (ER)-low expression (< 10%) tumors (combined: 14 out of 72; 19.4%; 95% CI 11.1–30.5). In contrast, we found concordant WBC and tumor methylation in only three out of 220 patients with 221 ER ≥ 10% tumors and zero out of 114 patients with 116 HER2-positive tumors. Intraindividually, *BRCA1* epimutations affected the same allele in normal and tumor cells. Assessing *BRCA1* methylation in umbilical WBCs from girls, we found mosaic, predominantly monoallelic *BRCA1* epimutations, with qualitative features similar to those in adults, in 113/1260 (9.0%) of individuals, but no correlation to *BRCA1* methylation status either in mothers or fathers. A significantly lower fraction of newborn boys carried *BRCA1* methylation (9/200; 4.5%) as compared to girls (*p* = 0.038). Similarly, WBC *BRCA1* methylation was found less common among fathers (16/531; 3.0%), as compared to mothers (46/575; 8.0%; *p* = 0.0003).

**Conclusions:**

Our findings suggest prenatal *BRCA1* epimutations might be the underlying cause of around 20% of TNBC and low-ER expression breast cancers. Such constitutional mosaic *BRCA1* methylation likely arise through gender-related mechanisms in utero, independent of Mendelian inheritance.

**Supplementary Information:**

The online version contains supplementary material available at 10.1186/s13073-023-01262-8.

## Background

Aberrant gene promoter methylation, or epimutations, is observed in many cancer types. While such epimutations may be passenger events of limited biological importance, it is well established that promoter methylation of tumor suppressor genes (TSGs) may contribute to tumor initiation and/or progression and play a significant role to tumor biology in general [1, 2].

Germline pathogenic variants (PVs) in the *BRCA1* and *BRCA2* genes are the most frequent cause of hereditary breast and ovarian cancers [3–5]. Most breast cancers arising in *BRCA1* PV carriers belong to the triple-negative subclass. Contrasting *BRCA2* [6], *BRCA1* is frequently methylated in sporadic TNBC and HGSOC tumors [7–9], and it is well established that such promoter methylation is associated with repressed *BRCA1* transcription [10, 11]. TNBCs with *BRCA1* methylation have a gene expression profile closely resembling the profile of TNBCs arising in *BRCA1* PV carriers [7]. While *BRCA1* promoter methylation and *BRCA1* PVs seem to a large extent to be mutually exclusive in both TNBCs and HGSOCs [7, 12], conflicting evidence indicates similarities and differences between tumors harboring *BRCA1* promoter methylation or a PV regarding therapy sensitivity in breast cancer [8, 13, 14].

Constitutional epimutations are defined as aberrant normal tissue methylation occurring in early life, generally affecting all three germ layers [15]. There are two types: secondary epimutations, caused by specific genetic aberrations, and primary epimutations, for which no underlying genetic factor is found [15]. Contrasting secondary epimutations, primary epimutations often present in a low-level, mosaic pattern, affecting only a small fraction of cells [12]. While secondary constitutional methylation of *BRCA1* has been observed in a few families with an elevated risk of breast and ovarian cancer [16–18], the question of primary constitutional methylation as a cancer risk factor has remained controversial [6, 12, 19–25]. However, in a recent study, we found white blood cell (WBC) *BRCA1* promoter methylation to predict an elevated risk of incident TNBC as well as HGSOC > 5 years after blood sampling in healthy women [26]. While these findings indicate that *BRCA1* methylation may arise in normal cells subsequently developing into cancer precursors, several key questions need to be addressed. First, we do not know whether WBC *BRCA1* mosaic methylation arise prenatally (constitutional) or may be acquired postnatally (somatic normal tissue methylation). In case of constitutional methylation, we need to address whether such methylation may be fully developed across the promoter prenatally or exists as an incomplete precursor for subsequent development at a later stage. Second, in case *BRCA1*-methylated cells arise by a prenatal clonal expansion as constitutional methylation, one would expect a qualitatively similar, allele-specific WBC methylation in newborns as that recorded in adults. Third, if *BRCA1*-methylated WBCs represent constitutional methylation, and *BRCA1*-methylated WBCs and breast cancer precursor cells share a common embryonic clonal origin, one would expect a similar allele-specific *BRCA1* methylation profile [26] in WBCs and matched *BRCA1*-methylated tumors from the same individual. Fourth, we need to assess the fraction of TNBCs arising from constitutionally *BRCA1*-methylated cells, i.e., the fraction of TNBCs, previously considered as “sporadic,” that could be explained by underlying *BRCA1* methylation. While we recently reported the hazard ratio for incident TNBC with respect to WBC *BRCA1* methylation to be 2.35 [26], such a hazard ratio provides an indirect estimate for the fraction of tumors actually derived by this mechanism [27]. Moreover, the fact that the median age of women enrolled in our previous study was 62 years, indicates that a substantial fraction of TNBCs may have been overlooked due to diagnosis at a younger age.

To address these questions, we evaluated the incidence, magnitude, intraindividual tissue concordance, and allele specificity of *BRCA1* methylation in tumor and matched WBC from 408 patients diagnosed with 411 primary breast cancers (three individuals harboring two synchronous tumors), including 66 TNBCs. In addition, we analyzed WBC *BRCA1* methylation in umbilical cord blood samples from 1260 newborn girls and 200 newborn boys. Furthermore, to explore a potential transgenerational transmission, we analyzed WBC samples from parents of newborns.

## Methods

### Patients and tissue sampling

**Fig. 1:**
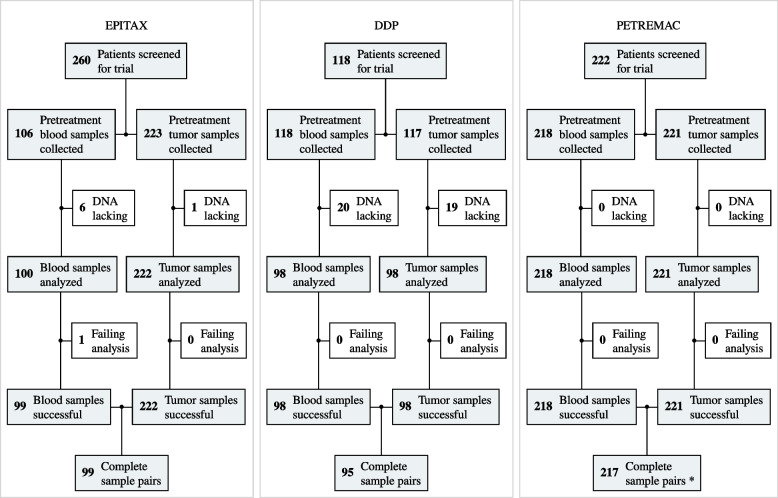
CONSORT diagram depicting patient enrolment in the EPITAX, DDP, and PETREMAC clinical trials, and the number of pretreatment samples collected and successfully analyzed in the current study. “*” symbol indicates the following: three patients were diagnosed with two synchronous primary tumors each: one patient diagnosed with two HER2-negative tumors expressing ER ≥ 10% and two patients diagnosed with two separate HER2 + tumors each

In the present study, we included all patients enrolled in three neoadjuvant breast cancer studies (EPITAX, DDP and PETREMAC) [8, 28, 29] from which pretreatment tumor tissue and WBC DNA samples were available for analysis (Fig. 1). The studies were approved by the Regional Ethics Committee (273/96–82.96, 06/3077 and 2015/1493), and all patients provided written informed consent at enrolment. The DDP and PETREMAC trials were registered under ClinicalTrials.gov (NCT00496795 and NCT02624973), while the EPITAX was conducted prior to ClinicalTrials implementation.

All patients underwent an incisional or Tru-Cut tumor biopsy prior to treatment. Tumor biopsies were snap-frozen in liquid nitrogen at removal and stored in liquid nitrogen, while WBC specimens were stored at –80 °C after centrifugation of EDTA whole blood and plasma removal.

The Norwegian Mother, Father and Child Cohort study (MoBa) is an ongoing cohort study enrolling more than 110,000 newborns and their parents [30]. For the present study, we randomly selected 200 newborn boys and 1260 newborn girls, including 420 girls born prematurely (before 36 weeks of gestation) and 840 girls born at normal term (39–41 weeks of gestation). The reason for selecting girls and a small number of boys only was based on previous unpublished data obtained with a methylation-specific PCR (MSP)-based method, indicating a gender difference with a higher frequency of *BRCA1* methylation among females compared to males. Procedures for sample collection, DNA extraction, and storage have been described previously [31]. No difference in *BRCA1* methylation was observed between premature and normal term newborns, and the samples were therefore treated as a unified cohort in the present study (Additional file 1, Fig. S1 and Table S1).

Finally, we analyzed *BRCA1* methylation among mothers and fathers of *BRCA1*-methylated newborn girls for whom DNA samples were available, together with parental samples from a random selection of > 400 *BRCA1*-nonmethylated newborn girls (Additional file 1, Fig. S1 and Table S2).

### Sample preparation

Procedures for DNA and RNA extraction from tumor and WBC samples are outlined in Additional file 1. In brief, genomic DNA for methylation analyses was extracted from tumor and WBC samples using QIAamp DNA Mini kit (Qiagen, Valencia, CA), and total RNA for gene expression analysis was extracted from tumor tissue using the RNeasy Mini kit (Qiagen, Valencia, CA).

### Methylation sequencing

For *BRCA1* methylation analysis, DNA bisulfite conversion, amplification, and sequencing was performed as described previously [26]. Briefly, 500 ng of genomic DNA were bisulfite converted and subjected to *BRCA1* gene promoter fragment amplification using four pairs of primers that do not overlap with any of the CpG dinucleotides (GRCh38 genomic coordinates: CpG00–13 chr17:43,125,624–43,126,026, CpG14–31 chr17:43,125,270–43125640, CpG17–34 chr17:43,125,171–43,125,550, CpG33–49 chr17:43,124,861–43,125,249; Additional file 1; Fig. S2). All four amplicons were combined, indexed, and sequenced by 2 × 226 bp reads using Illumina MiSeq System (Illumina, San Diego, CA), resulting in an ultradeep coverage of about 30,000 × for each amplicon. As previously described for case–control analyses [26], the region covering CpGs 14–34 is considered biologically relevant and was used as the main measure for methylation calling. The overlapping amplicon covering CpGs 33–49 also covered SNP rs799905 and was used for allele-specific methylation assessment.

### Tumor molecular subtyping

Estrogen and progesterone receptor (ER, PgR) as well as HER2 status were determined upon inclusion in each of the clinical trials. For the present analyses, we defined the cutoff for ER positivity as 1%. Since the EPITAX trial, conducted in 1997–2003, used a cutoff of 10%, all cases where ER status was recorded as < 10% were re-examined according to standard criteria and classified as either < 1% or 1–9%.

All tumors were assigned to intrinsic subtypes based on mRNA expression profiling according to the classification by Perou et al. [32] using either RNA sequencing (DDP and PETREMAC trials) or mRNA microarrays (EPITAX trial) (for details, see Additional file 1).

### *BRCA1* PV assessment

Data on *BRCA1* PV status for patients were collected from our previous genetic analyses [8, 29]. For cases lacking previous genetic data, we performed targeted sequencing of a cancer gene panel, as previously described [33], and drew *BRCA1* PV status from the generated data. For consistency, all detected variants were re-audited for pathogenicity according to the ClinVar database [34] on April 20, 2023.

### Data analyses

For *BRCA1* methylation analysis, sequencing reads were mapped/aligned to the GRCh38 reference genome using the Illumina DRAGEN Bio-IT Platform (v3.6.3). Cytosine methylation and its allele specificity was evaluated using the epialleleR R package (v1.3.5) [35]. A single quantitative metric of methylation (hypermethylated variant epiallele frequency, VEF) was obtained by averaging frequencies of hypermethylated epialleles for two amplicons covering CpGs 14–31 and 17–34 as previously reported [26]. The cutoffs for methylation positivity were determined computationally, following the same predefined approach as previously reported [26] (for details, see Additional file 1, Figs. S3–S6). The cutoffs were defined as 6.96 × 10^−4^ for *BRCA1* methylation in WBC and 4.71 × 10^−2^ for tumors. The differences between the cutoffs in WBC and tumors also reflect a biological rationale: WBC *BRCA1* methylation is expected to present a low-level, mosaic pattern [12, 26]. In contrast, assuming clonal expansion, tumors arising from *BRCA1*-methylated cells should be expected to harbor a larger fraction of methylated cells.

### Statistical analysis

Concordance in methylation status between tumor and corresponding WBC samples, and differences in methylation incidence between newborn girls and boys as well as between adult females and males were all compared using the Fisher’s exact test. Methylation frequencies were presented with confidence intervals. Methylation levels (VEF) in newborns (girls and boys) and young adults (fathers and mothers) were compared using a two-way analysis of variance, while VEF values of cancer patients and healthy individuals (newborns and parents) were compared using Student’s paired *t*-test. R software environment for statistical computing (v4.1.2) was used for all statistical analyses.

## Results

### Study population characteristics

To assess *BRCA1* methylation in breast cancer patients, we included all patients enrolled in three neoadjuvant breast cancer studies (the EPITAX, DDP, and PETREMAC trials) [8, 28, 29] from whom pretreatment tumor tissue and matched WBC DNA samples were available. Among a total of 600 patients screened, 408 had both blood (*N* = 408) and tumor (*N* = 411) pretreatment samples successfully analyzed and were included in the final results. Of these, three patients from the PETREMAC study were diagnosed with two primary tumors each: one patient with two HER2-negative tumors expressing ER ≥ 10% and two patients with two HER2 + tumors each. These six tumors were treated as separate events in all downstream analyses (Fig. 1). No statistically significant differences in the distribution of tumor molecular subtypes and receptor status between patients included in the three trials were recorded (Table 1).

**Table 1:** Patient characteristics in the EPITAX, DDP and PETREMAC clinical trials

	**EPITAX** **(N = 99)**	**DDP** **(N = 95)**	**PETREMAC** **(N = 217)**
**Age**			
Mean (SD)	49.5 (10.3)	48.4 (9.80)	53.0 (10.9)
Median [Min, Max]	49.0 [25.0, 70.0]	48.0 [24.0, 71.0]	51.0 [27.0, 78.0]
Tumor receptor status *			
TNBC	16 (16.2%)	17 (17.9%)	33 (15.2%)
HER2–/ER<10%	4 (4.0%)	0	2 (0.9%)
HER2–/ER≥10%	48 (48.5%)	59 (62.1%)	114 (52.5%)
HER2+	29 (29.3%)	19 (20.0%)	68 (31.3%)
Missing	2 (2.0%)	0	0
Tumor molecular subtype *			
Basal	24 (24.2%)	14 (14.7%)	29 (13.4%)
Her2	20 (20.2%)	14 (14.7%)	45 (20.7%)
LumA	22 (22.2%)	32 (33.7%)	70 (32.3%)
LumB	22 (22.2%)	25 (26.3%)	51 (23.5%)
Normal	11 (11.1%)	10 (10.5%)	20 (9.2%)
Missing	0	0	2 (0.9%)
**Tumor histology**			
IDC	80 (80.8%)	72 (75.8%)	159 (73.3%)
ILC	15 (15.2%)	17 (17.9%)	32 (14.7%)
Other	4 (4.0%)	6 (6.3%)	26 (12.0%)

* N* number of tumor samples, *TNBC* triple-negative breast cancer, *HER2* Human epithelial-like receptor-2, *ER* estrogen receptor, *basal* basal-like gene expression profile, *Her2* HER2-enriched gene expression profile, *LumA* luminal A gene expression profile, *LumB* luminal B gene expression profile, *Normal* normal-like gene expression profile, *IDC* invasive ductal carcinoma, *ILC* invasive lobular carcinoma

^*^Test of heterogeneity between cohorts (Chi-Square): *p* > 0.2

To assess allele-specific mosaic *BRCA1* methylation in newborns, umbilical cord blood samples from 1260 girls and 200 boys were drawn from the Norwegian Mother, Father and Child Cohort study (MoBa) [30], as listed in Additional file 1; Fig. S1 and Table S1. After analyzing these umbilical cord blood samples, available blood samples from both parents of *BRCA1-*methylated girls together with samples from a random selection of parents of *BRCA1-*nonmethylated girls were collected and analyzed for *BRCA1* methylation (Additional file 1; Fig. S1 and Table S2). The samples from parents were analyzed blinded to methylation status of the newborn.

### Concordant WBC and tumor *BRCA1* methylation

**Fig. 2:**
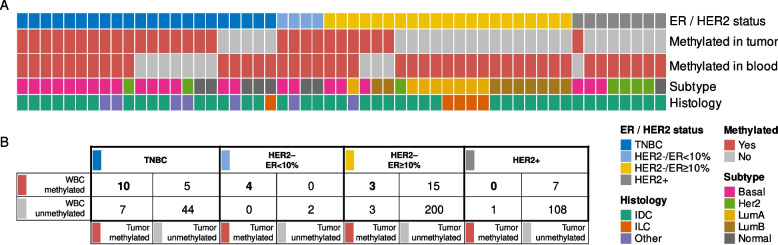
*BRCA1* methylation in matched blood and tumor samples in breast cancer patients. **A** Molecular and histological characteristics (rows) of all samples (*N* = 55; columns) belonging to matched sample pairs carrying *BRCA1* methylation in the blood (WBC) and/or tumor. TNBC, triple-negative breast cancer; HER2, Human epithelial-like receptor-2; ER, estrogen receptor; basal, basal-like gene expression profile; Her2, HER2-enriched gene expression profile; LumA, luminal A gene expression profile; LumB, luminal B gene expression profile; Normal, normal-like gene expression profile; IDC, invasive ductal carcinoma; ILC, invasive lobular carcinoma. **B** Concordance of *BRCA1*-methylation status in WBC and tumor tissue among all patients analyzed, stratified for tumors belonging to the different breast cancer subgroups

Based on the previously detected association between constitutional *BRCA1* methylation and risk of TNBC, we analyzed concordance of *BRCA1* methylation in tumors and matched WBC samples to assess the fraction of TNBCs potentially caused by underlying constitutional *BRCA1* methylation. In total, 17 out of 66 (25.8%; 95% CI 15.8–38.0) patients with TNBC harbored tumor *BRCA1* methylation. Notably, among these 17 patients, 10 (58.9%; CI 32.9–81.6%) also carried WBC *BRCA1* methylation (WBC and tumor tissue methylation concordance: *P* < 0.001; Fig. 2; Additional file 1, Table S3). Thus, 15.2% (95% CI 7.5–26.1%) of all TNBCs revealed concordant tumor and WBC *BRCA1* methylation. As for patients with *BRCA1*-unmethylated TNBC tumors, 5 out of 49 (10%; 95% CI 3.4–22.2%) harbored WBC *BRCA1* methylation (Fig. 2).

Regarding tumors with ER expression within 1–9%, genomic profiling has revealed these tumors to mirror gene expression profiles recorded in TNBC [36]. In this subgroup, four out of six patients harbored *BRCA1* methylation in their tumor tissue, all revealing concordant WBC methylation (Fig. 2). Grouping the TNBC and ER-low (1–9%) tumors together, concordant tumor and WBC *BRCA1* methylation was observed in 14 out of 72 patients (19.1%; 95% CI 11.1–30.5%). Notably, these 14 constituted the majority of the 21 patients with *BRCA1* methylated TNBC or ER low (1–9%) tumors (66.7%; 95% CI 43.0–85.4%).

For HER2-negative tumors expressing ER ≥ 10%, six out of 221 revealed *BRCA1* tumor methylation with only three of these patients revealing concordant WBC *BRCA1* methylation. Furthermore, the lowest methylation frequency was observed among HER2-positive tumors (independent of ER expression). Here, one out of 116 tumors revealed *BRCA1* methylation in the tumor tissue, and this patient was negative for WBC *BRCA1* methylation (Fig. 2). None of the patients with two primary tumors revealed either tumor or WBC *BRCA1* methylation, and none of these tumors were either TNBC or ER low expressing tumors.

**Fig. 3:**
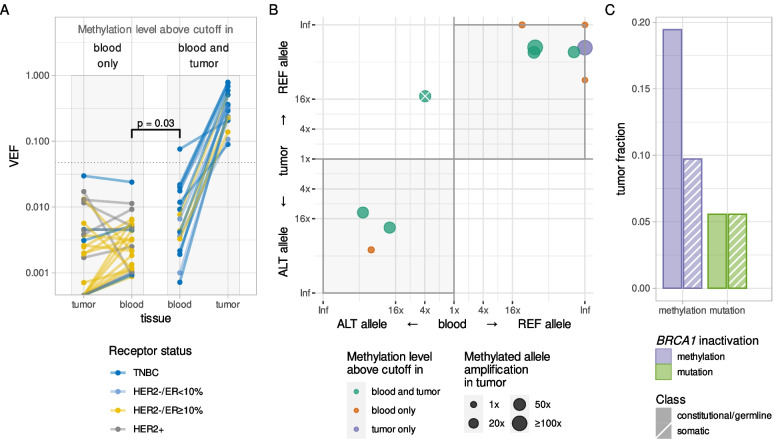
**A** Quantitative levels of *BRCA1* methylation (VEF value for region CpG14–34) in blood and tumor samples of breast cancer patients from whom blood samples had *BRCA1* methylation levels above the blood-specific cutoff. Solid lines connect matched samples; lines and dots are colored according to tumor receptor status. Gray boxes outline patients with *BRCA1* methylation *not enriched* (left) or *enriched* (right) in tumors; dotted line represents cutoff value for *BRCA1* methylation positivity in tumor tissue. Both quantitative (ANOVA) and qualitative (Wilcoxon rank sum) tests confirm significant difference between subsets of blood VEF values (shown by square bracket).) Allele specificity of *BRCA1* methylation in blood and tumor samples from breast cancer patients heterozygous for SNP rs799905 (*N* = 11). Preferential methylation of one of the alleles is evaluated and plotted as fold enrichment, with allele-specific preference in methylation in blood on the *x*-axis and in tumor on the *y*-axis. Gray shading indicates quadrants supporting concordant allelic methylation in matched blood and tumor. Data points falling in the upper-right quadrant indicate the reference allele of rs799905 to be the predominantly methylated allele in blood and tumor, while data points falling in the lower-left quadrant indicate the alternative allele of rs799905 to be the predominantly methylated allele in blood and tumor. Dots (representing matched sample pairs) are colored according to *BRCA1* methylation status in tumor and blood with their size representing fold amplification of the methylated allele in tumor tissue compared to the corresponding blood sample. The crossed-out dot represents an individual with comparable methylation of both alleles in blood but predominantly the reference-allele methylated in tumor, likely reflecting the tumor to have originated from one out of two methylated lineages of normal cells (see main text). Inf, infinity value, i.e., exclusive methylation of a single allele. **C** Fractions of TNBC and HER2–/ER < 10% tumors (*N* = 72) characterized by different molecular mechanisms of *BRCA1* inactivation (methylation [blue] or mutation [green]) and its potential time of emergence (constitutional/germline [solid fill] or somatic [stripe pattern])

As for patients harboring *BRCA1* methylation in both tumor and WBC, *BRCA1* methylation levels in the tumors were 36–103 fold higher than in blood, consistent with clonal expansion of cells with methylated *BRCA1* alleles (Fig. 3A). Intratumoral levels of *BRCA1* methylation were similar for TNBCs (7.6–78.8%), ER-low (10.7–36.4%), and the remaining non-TNBC methylated tumors (13.8–82.4%). Details regarding methylation levels (VEF) across *BRCA1*-methylated tumor and WBC samples, demographic data on patients with *BRCA1*-methylated tumors and/or WBCs, and treatment responses related to tumor *BRCA1* methylation are presented in Additional file 1, Figs. S7 and S8, and Tables S3 and S4.

Taken together, these findings indicate that around 20% of all TNBC/ER-low breast cancers and around 65% of all *BRCA1*-methylated tumors occur in individuals with underlying constitutional *BRCA1* methylation.

### Allele-specific concordance of *BRCA1* methylation in WBC and tumor tissue

While concordant *BRCA1* methylation in tumor tissue and matched blood samples may suggest a common clonal origin, we sought to provide further evidence for this hypothesis by assessing the allele specificity of the *BRCA1* methylation in tumors and blood.

Allele-specific methylation may be detected in cases heterozygous for the SNP rs799905, since this polymorphism is located in the area that is covered by the *BRCA1* methylation assay (see Methods and Additional file 1, Fig. S2). Among 17 patients carrying concordant *BRCA1* methylation in WBC and tumor tissue, SNP rs799905 genotype information was lacking and/or could not be linked to methylation in two patients. For the 15 informative individuals, seven were homozygous for the reference allele, two were homozygous for the alternative allele, while six were heterozygous. The allelic distribution of *BRCA1* methylation for WBCs and tumor samples among these six informative heterozygous cases is depicted in Fig. 3B (green dots). *BRCA1* methylation was enriched on the same allele in the tumor tissue and WBC in five of these individuals, indicating a shared clonal origin of the methylated normal and tumor cells. The sixth patient revealed comparable levels of *BRCA1* methylation of both rs799905 alleles in blood (with a slight preference for methylation on the alternative allele), while the tumor carried methylation of the reference allele. Most likely, this patient harbored two independent subclones of *BRCA1*-methylated normal cells, with one clone giving rise to the tumor cells.

As the tumor samples were not subject to microdissection, they contain a number of different types of benign cells including normal breast epithelium, fibroblasts, circulating WBCs, and macrophages [37]. Among individuals harboring WBC but not tumor *BRCA1* methylation (*n* = 27), 17 revealed small traces of *BRCA1*-methylated cells in the tumor biopsies, below the defined threshold for classification of tumors as methylation-positive but above the methylation threshold applied to WBC samples. This is consistent with low-level mosaic *BRCA1* methylation in normal breast cells and/or other normal cells present in the tumor biopsies. Among these 17 patients, four were heterozygous for rs799905 and thus informative for allele-specific methylation status. These four all revealed the low-level *BRCA1* methylation in their tumor biopsies to share the same magnitude and allele specificity as the methylation in the matched WBCs (Fig. 3B, red dots).

In addition, one patient with a *BRCA1* methylated TNBC and WBC *BRCA1* methylation just below the formal cutoff for positivity could be assessed for allelic methylation concordance. This patient also revealed a similar allele specific *BRCA1* methylation in tumor and WBC samples (Fig. 3B, blue dot).

Taken together, these findings reveal an allelic concordance between *BRCA1* methylation in WBC and matched cancer or benign tissue in the breast cancer samples, indicating that the methylated tumors have arisen from methylated normal cells in the affected mosaic individuals.

### Intrinsic breast cancer subtypes and *BRCA1 *methylation

The distribution of *BRCA1* methylation was determined among the intrinsic subtypes of breast cancers, based on their mRNA signatures [32]. The subtype distribution of *BRCA1-*methylated tumors did not differ between TNBCs and non-TNBCs, neither was there a difference between those tumors harboring concordant WBC *BRCA1* methylation and those that did not (Fig. 2A; Additional file 1, Fig. S9). Regarding TNBCs and ER < 10% tumors with concordant tumor and WBC *BRCA1* methylation, 11 out of 14 were basal-like, two were normal-like, while one tumor expressed a HER2-enriched profile, despite absence of *HER2* gene amplification or positive protein staining. Interestingly, among the three ER ≥ 10% tumors revealing concordant tumor and WBC *BRCA1* methylation, two revealed a basal-like profile, while the remaining one was classified as luminal A.

### *BRCA1* PVs and methylation

Among the 411 tumors analyzed, nine harbored *BRCA1* pathogenic variants (four somatic and five germline; Fig. 3C; Additional file 1, Table S5). None of the patients with germline *BRCA1* pathogenic variants revealed either WBC or tumor *BRCA1* methylation. While one patient with a somatic *BRCA1* PV harbored tumor tissue *BRCA1* methylation in concert, no *BRCA1* methylation was detected in this patient’s WBCs, indicating the tumor *BRCA1* methylation, similar to the PV, to be a somatic event.

### Frequency and allele specificity of *BRCA1* methylation in WBC from newborns

**Fig. 4:**
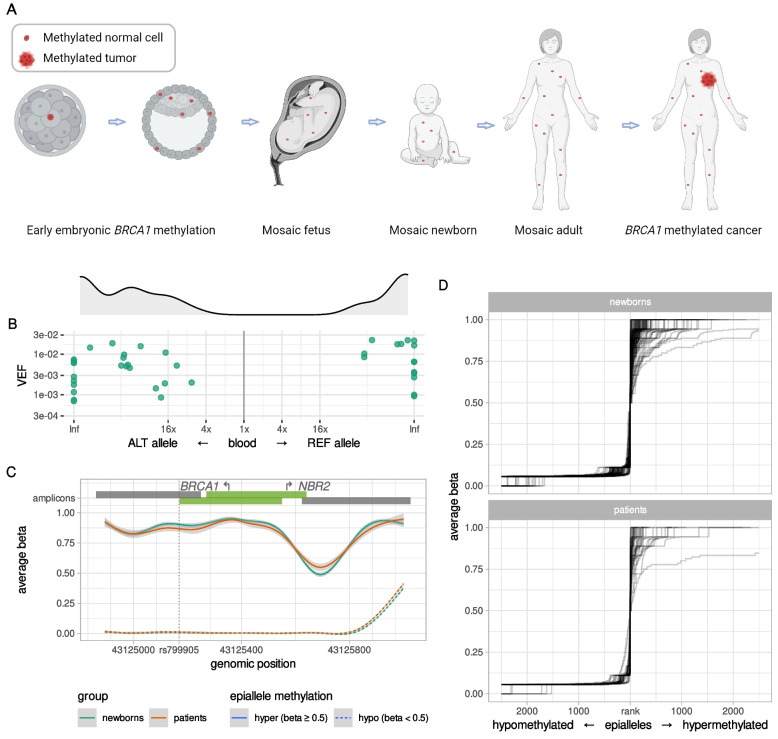
Similar properties of *BRCA1* methylation in blood samples of healthy newborn girls and adult breast cancer patients. **A** Overall model for early prenatal (constitutional) *BRCA1* methylation as an underlying contributor to TNBC. Red dots represent *BRCA1*-methylated normal cells, appearing through an early embryonic event, resulting in a mosaic adult. Red star represents breast cancer. **B** Allele specificity of *BRCA1* methylation in blood samples from newborn girls heterozygous for SNP rs799905 (*N* = 40). Preferential methylation of one of the alleles (fold enrichment) is indicated on the *x*-axis and degree of methylation (VEF value for region CpG14–34) is indicated on the *y*-axis. Data points in the right half of the plot indicate methylated alleles to be predominantly rs799905 reference alleles, while data points towards the left indicate methylated alleles to be predominantly rs799905 alternative alleles. Gray area above the plot shows smooth kernel density estimates for fold enrichment values. Inf, infinity value, i.e., exclusive methylation of a single allele. **C** Smoothed averaged CpG methylation levels (*y*-axis) within assayed genomic region (*x*-axis) in blood of *BRCA1* methylation-positive newborn girls (*N* = 113; green lines) and breast cancer patients (*N* = 44; red lines). Solid lines represent averages for all hypermethylated epialleles (per-epiallele average beta value ≥ 0.5); dashed lines represent averages for all hypomethylated epialleles (per-epiallele average beta value < 0.5); light gray areas represent 95% CI. Bars on top represent amplicons, with the bright green ones covering CpGs 14–34. Arrows show *BRCA1* and *NBR2* transcription start sites; vertical dotted line marks position of SNP rs799905 (see Supplementary Information for more details). **D** Average beta values (*y*-axis) of ranked epialleles (*x*-axis) in blood samples of *BRCA1* methylation-positive newborn girls (*N* = 113; top) and breast cancer patients (*N* = 44; bottom). All epialleles of the region CpG14–34, within each sample, were ranked by increasing average beta value with every rank centered at epiallele with average beta value of 0.5. Lines connect increasing beta values and represent individual samples. Maximum 5000 epialleles are plotted per sample (beta = 0.5, ± 2500 alleles). The sharp incline in average beta value around beta = 0.5 reveals that most alleles are either hypomethylated or hypermethylated; very few alleles have intermediate methylation levels

To assess the hypothesis of early origin (Fig. 4A) and potential dynamics of *BRCA1* epimutations and their allele specificity, we analyzed umbilical cord blood samples from 1260 newborn girls and 200 newborn boys. We found 113 out of 1260 newborn girls to be *BRCA1* methylation-positive (9.0%; 95% CI 7.5–10.7%). Among the 113 newborn girls revealing WBC *BRCA1* methylation, sufficient amounts of DNA for SNP rs799905 assessment were available from 89. Out of these, 40 were heterozygous for SNP rs799905 and therefore evaluable for allele specificity of the methylation. Intraindividually, methylation was located predominantly on one specific allele with an equal distribution between the two genotypes (Fig. 4B; *p* > 0.10). Moreover, the average intramolecular methylation pattern seen in newborns were indistinguishable from the ones in adults (Fig. 4C; Additional file 1, Fig. S10), both sharing a relatively sharp distinction between non-methylated and hypermethylated epialleles, with the majority of methylation-positive epialleles being close to fully methylated (i.e., methylated on all CpGs), and very few epialleles having intermediate methylation levels (i.e., methylated at 20–80% of CpGs; Fig. 4D; Additional file 1, Fig. S10). These findings reveal a methylation pattern similar to that seen in adult cancer patients, indicating a clonal origin.

*BRCA1* methylation occurred at a significantly lower frequency in boys with only nine out of 200 (4.5%; 95% CI 2.1–8.4%) carrying WBC *BRCA1* methylation (frequency compared to girls: *p* = 0.038).

**Fig. 5:**
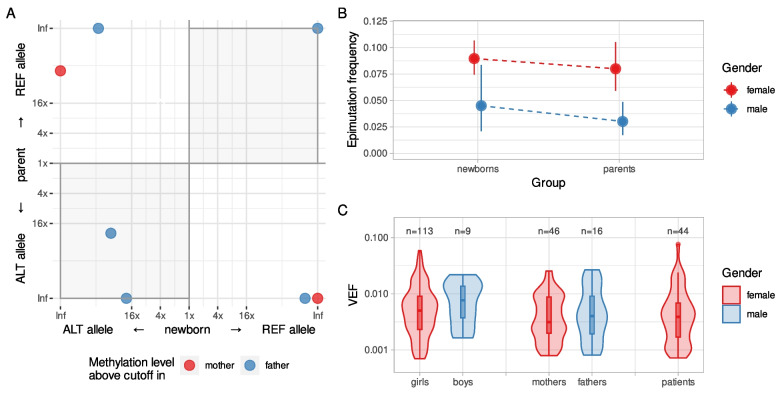
Properties of *BRCA1* methylation in blood samples of newborns and their parents. **A** Allele specificity of *BRCA1* methylation in blood samples of newborns and their parents (*N* = 7). Preferential methylation of one of the alleles is evaluated and plotted as fold enrichment, with allele-specific preference in methylation in blood of newborns on the *x*-axis and in blood of parents on the *y*-axis. Gray shading indicates quadrants supporting concordant allelic methylation within families. Data points falling in the upper-right quadrant indicate the reference allele of rs799905 to be the predominantly methylated allele in both samples, while data points falling in the lower-left quadrant indicate the alternative allele of rs799905 to be the predominantly methylated allele in both samples. Dots (representing matched sample pairs) are colored according to the gender of the *BRCA1*-methylated parent. **B** Fractions of *BRCA1* epimutation carriers among newborns and their parents. Fractions (epimutation frequencies) are plotted as dots, colored according to gender. Dashed lines connect age groups of the same gender. Solid vertical lines represent 95% CI. **C** Density plot of *BRCA1* methylation levels (VEF value for region CpG14–34) in blood samples of newborn boys and girls (*N* = 9 and 113, respectively), their fathers and mothers (*N* = 16 and 46, respectively), and breast cancer patients (*N* = 44), colored according to gender. The lower and upper hinges of boxes correspond to the first (Q_1_) and third (Q_3_) quartiles, respectively; the bar in the middle correspond to the median value; the upper and lower whisker extend to Q_3_ + 1.5*IQR and Q_1_–1.5*IQR, respectively, while the values outside this range (outliers) are plotted as dots. Neither quantitative (ANOVA) nor qualitative (Wilcoxon rank sum) test show significant difference between any of these sets of blood VEF values

To assess potential inheritance, *BRCA1* methylation concordance among the newborn girls and their mothers or fathers was analyzed (Additional file 1, Fig. S1 and Table S2). Notably, there was no significant association between methylation status among newborn girls and either maternal or paternal *BRCA1* methylation status (Table 2). Furthermore, assessing allele-specific methylation among methylation-positive girls and their methylation-positive parents for whom both were informative for SNP rs799905 status (*n* = 7) revealed no indication of inheritance, with concordant allele methylation in three but discordant allele methylation in four pairs (Fig. 5A; Additional file 1, Table S6).

**Table 2:** *BRCA1* methylation concordance in newborns and their parents

		Fathers *		Mothers *		Any parent*	
		M	U	M	U	M	U
Newborn girls *	M	5	88	6	96	11	91
		(1%)	(17%)	(1%)	(17%)	(2%)	(16%)
	U	11	427	40	433	51	423
		(2%)	(80%)	(7%)	(75%)	(9%)	(73%)

^*^M and U, *BRCA1* methylation positive and *BRCA1* methylation negative, respectively. *p* > 0.1 for all comparisons for concordance

Similar to the gender difference among newborns, *BRCA1* methylation occurred at a significantly lower frequency in adult males (fathers) as compared to females (mothers) with 16 out of 531 fathers (3.0%; 95% CI 1.7–4.8%) versus 46 out of 575 mothers (8.0%; 95% CI 5.9–10.5%) (*p* = 0.0003; Fig. 5B) carrying *BRCA1* methylation.

Notably, methylation levels (VEF) of individuals carrying *BRCA1* methylation did not differ between newborn boys and girls and healthy adult males and females (two-way analysis of variance: *p* > 0.1 for all comparisons; Fig. 5C). Furthermore, no difference in VEF between newborns or parents, single groups or combined, on the one hand, and cancer patients on the other hand, was recorded, and the VEF in all groups of healthy individuals and cancer patients were within a similar range (Fig. 5C).

## Discussion

Epigenetic regulation plays a key role in normal cell function during life and is influenced by genetic as well as environmental factors. Epimutations may occur either as somatic events during life or as so-called constitutional methylation, arising in utero, in which case it may affect normal tissues derived from all three germ layers [15]. While epimutations causing gene silencing of tumor suppressors like *MGMT*, *MLH1*, and *BRCA1* are well known across many malignancies [8, 10, 38–41], in general, such epimutations are considered somatic. However, the seminal discovery by Gazzoli in 2002 [42] followed by the work of Hitchins and colleagues [43] revealing constitutional *MLH1* methylation as a trigger in colon cancer sparked interest in constitutional methylation of TSG promoters as a potential underlying cause of cancer [15, 44]. Subsequent studies revealed at least some of these *MLH1* epimutations to be secondary to genetic aberrations [45].

While WBC *BRCA1* methylation has been associated with an elevated hazard ratio of triple-negative breast cancer, to this end, the quantitative contribution of normal tissue *BRCA1* methylation to TNBC and, potentially, non-TNBC, has remained unknown due to lack of studies evaluating concordant *BRCA1* methylation in tumor tissue and matched WBC. Analyzing *BRCA1* methylation in matched blood and tumor tissue of patients with both TNBC and non-TNBC, we found a strong correlation between tumor tissue and WBC *BRCA1* methylation in TNBC and tumors revealing a low ER expression (1–9%). In this group, 29.2% of tumors were *BRCA1*-methylated, and 19.4% harbored concordant tumor and WBC methylation. While no previous data exist regarding the incidence of concordant *BRCA1* methylation in tumor and WBCs, our findings for tumor methylation in total (somatic plus constitutional origin) aligns with the finding by Glodzik et al. [7] of 30% of TNBCs carrying tissue *BRCA1* promoter methylation. Importantly, if a percentage as high as 20% of TNBCs/ER-low breast cancers having concordant WBC methylation is reproduced across other cohorts, this would mean that a substantially larger fraction of TNBCs may be caused by constitutional methylation than by pathogenic germline variants.

In contrast, among breast cancers with HER2 overexpression or ER levels of ≥ 10%, constitutional *BRCA1* tumor methylation was a rare event. However, interestingly, two out of three such tumors in patients with constitutional methylation revealed a basal-like gene expression signature.

Recently, we reported WBC *BRCA1* methylation to be predominantly monoallelic, enriched on the same allele across the vast majority of normal blood cells in affected adult individuals [26]. Here, we significantly extended this observation by showing concordant allele-specific methylation in normal and malignant cells in our cancer patients. This is consistent with a common clonal origin of methylated normal and cancer cells, which supports the hypothesis that these *BRCA1*-methylated tumors have arisen from *BRCA1*-methylated normal cells.

In addition, in four patients informative for methylation allele specificity in WBC, we found the tumor samples to reveal low-level *BRCA1* methylation, likely reflecting a fraction of benign cells in the biopsy. Here, we found similar allelic concordance for *BRCA1* methylation in WBC and the presumed normal breast tissue. While such low-level methylation in theory could reflect small subclones of somatically methylated tumor cells, the chance of allelic concordance across four paired datasets is < 7%. In contrast, the finding of similar allele-specific methylation in WBC and normal breast tissue is what one would expect in cases of constitutional (prenatal) methylation. While the term “non-malignant cells” in a breast cancer biopsy covers different cell types of ectodermal as well as mesenchymal origin (ductal lining, immune cells, mesenchymal cells etc.) [37], constitutional methylation generally affects cells derived from all three embryonic germ layers [15].

While our patient data are consistent with constitutional *BRCA1* methylation, a pivotal question is whether normal tissue *BRCA1* methylation develops as a complete promoter methylation (effectively repressing *BRCA1* expression) in utero or arise as a partially functional epimutation, subsequently developing into complete promoter methylation in normal cells postnatally. Previous studies by us and others reporting WBC *BRCA1* methylation among newborn girls [12, 46] assessed methylation status by conventional analyses (MSP) preventing detailed assessment of allele specificity and quantitative characteristics of methylation. Notably, there is evidence showing that gene promoter methylation may develop in a stepwise manner [47]; thus, methylation previously recorded by MSP in newborns could present a premethylation step or a qualitatively different methylation process from the one detected in normal cells of adults. In the present study, we found *BRCA1* methylation in newborn girls to qualitatively and quantitatively mirror the one seen in adult cancer patients and previously recorded in healthy adults [26], indicating that the methylation observed in newborns and in adults is the same molecular feature.

Taken together, these findings, in concert with our findings of similar allelic methylation status in WBC and tumor tissue in adults, are consistent with a common clonal origin of all *BRCA1*-methylated cells within each patient, indicating that methylation may have arisen as a single-cell event during early embryonic development with subsequent clonal expansion across all germ layers.

While the mechanisms behind prenatal *BRCA1* mosaic epimutations remains unknown, we found no correlation between newborn and parental *BRCA1* methylation status, indicating Mendelian heritage to play a minor, if any, role. Furthermore, for those cases in which allele specificity of methylation in both the newborn and parents could be assessed, no concordance between parental and newborn allele-specific methylation was recorded. Taken together, these findings indicate that normal tissue *BRCA1* methylation may arise as an early prenatal somatic event generating methylated subclones, resembling recent findings in respect to cancer-promoting embryonic PVs [48, 49].

A most striking finding was the difference in methylation frequency between females and males, recorded both among newborns as well as young adults. This indicates the mechanism behind *BRCA1* methylation to be gender-dependent.

Analyzing WBCs collected from patients diagnosed with their breast cancer, it is important to exclude the possibility that WBC methylation is due to contamination from the tumor, either as circulating tumor DNA or circulating tumor cells. Yet, while the possibility of tumor contamination cannot be excluded for each case, it is unlikely to be the cause of the WBC *BRCA1* methylation observed for several reasons. First, considering the number of circulating tumor cells, even among patients with a substantial cancer burden, such cells account for less than one in a million cells [50]. Second, as for circulating tumor DNA [51, 52], the plasma volume currently required for detection of tumor-derived genomic aberrations in blood samples is far above any possible plasma remnants in our WBC assay. Finally, in our recent study [26], we confirmed WBC *BRCA1* methylation in healthy women to predict subsequent incident TNBC as well as HGSOC > 5 years after sampling, providing proof for normal cell *BRCA1* methylation to be a precursor for TNBC and HGSOC. Notably, WBC methylation levels (VEF) did not differ between individuals diagnosed with cancer and healthy newborns or adults. On the contrary, the possibility exists that for some patients harboring *BRCA1* methylation in the tumor tissue but not in WBC, methylation in the WBCs below the sensitivity of our assay may be present. While the sensitivity of our method (detecting *BRCA1* epimutations at the frequency below 10^−3^) exceeds the sensitivity of other methods in current use (like array-based methods and MSP), biologically relevant methylation may also be present at levels below what we may currently detect.

Regarding the patients with detected WBC *BRCA1* methylation, one may assume that the risk of TNBC would be correlated with the methylation level (VEF). In the present data set, however, the number of patients carrying *BRCA1* WBC and tumor methylation in concert does not allow for assessment of such a potential correlation.

Cancer patients may have a different WBC subfraction composition as compared to healthy individuals. Thus, a potential uncertainty in the present study relates to cancer-related changes in the WBC subfraction composition. While global methylation patterns vary between leukocyte subfractions [53, 54], examining *BRCA1* methylation status across previously reported datasets from adults [55], newborns, and corresponding 5-year-old children [31, 56], we detected no difference in *BRCA1* methylation status between the different WBC subfractions [12]. Thus, the observed differences in *BRCA1* methylation may not be a consequence of differences in WBC subfraction composition.

Taken together, we consider these findings to validate and justify the use of WBC *BRCA1* methylation as a marker of constitutional methylation in most individuals, including patients diagnosed with their primary breast cancer.

In our recent case–control WHI study [26], WBC *BRCA1* methylation was associated with an increased risk of incident TNBC (hazard ratio, HR 2.5) and HGSOC (HR 1.8). Notably, in that study, the median age at inclusion was 62 years, yet, TNBCs are known in general to be detected at an earlier age compared to other breast cancer subtypes [7]. Regarding HGSOC, the risk estimates in the WHI study is lower than that previously observed by us (HR 2.2–2.9) in a hospital-based cohort study in Norwegian women in which methylation was assessed by MSP [12]. Thus, the possibility exists that the lifetime risk for TNBC is higher than what we recorded in the WHI study. While the number of cases in the present study is limited, our finding that constitutional *BRCA1* methylation may account for 19.4% of all triple-negative and ER-low breast cancers is high given the observed *BRCA1* methylation frequency in the population (5.6% among non-cancer females in the US [26], 8.0% among young mothers and 9.0% among healthy newborns in the current study). Since constitutional *BRCA1* methylation affects a small fraction of normal cells in the individual, one may assume that the background incidence of *BRCA1* unmethylated breast cancers (including TNBC) is similar among carriers and non-carriers of *BRCA1* constitutional methylation. Based on this, our present data indicates that constitutional *BRCA1* methylation could be associated with a hazard ratio perhaps as high as 3–5 for TNBC/ER-low BC development [27].

In summary, we find constitutional *BRCA1* methylation, as defined by WBC methylation, to be linked to TNBC/ER-low BC. As for patients diagnosed with TNBC or ER-low tumors, our findings indicate that *BRCA1* promoter methylation should be explored as a potential risk factor for subsequent cancer development. Moreover, comparing cancers carrying the methylation on a constitutional versus somatic background should be performed to fully elucidate potential pathogenic consequences. The presence of *BRCA1* methylation on the same *BRCA1* allele in WBC and breast cancer DNA in the same patients adds strong support to the hypothesis that *BRCA1*-methylated tumors may arise from constitutionally *BRCA1-*methylated normal cells, likely initiated as an early, prenatal event [15]. Thus, our findings conceptually differ from normal tissue global methylation signatures designed for early cancer detection [57–59].

Our findings presented here, in concert with our recently published data [26], indicate that *BRCA1* methylation occurs as an early somatic embryonic event, affecting nearly 9% of newborn girls, and is associated with a substantial elevated cancer risk. This urges for further research identifying the potential causes of mosaic epimutations. To this end, cancers have generally been classified in two main groups: those arising on a background of pathogenic germline variants and those regarded as spontaneous tumors, with a grey zone of tumors due to low-risk variants and genes, in-between. Our findings question whether mosaic constitutional methylation of other tumor suppressor genes, beyond *BRCA1*, could be a significant risk factor to other cancer forms as well. While a limited number of cancer cases associated with constitutional *MLH1* methylation have been reported [44, 60, 61], screening newborns for constitutional *MLH1* epimutations we found this to be a rare event (< 0.1%; Nikolaienko et al.; unpublished observations). Still, constitutional methylation of the vast majority of tumor suppressor genes remains unexplored, and it may be that prenatal epimutations in such genes are pan cancer risk factors. As such, the present findings may point toward the dawn of a new era, suggesting that a substantial number of cancers may develop from prenatally epimutated cell clones.

## Conclusions

In this study, we show intraindividual allele concordance between mosaic *BRCA1* methylation in normal blood cells and *BRCA1* methylation, clonally expanded, in tumor tissue. This is consistent with the methylation event occurring at an early embryonic stage. Our data suggest that about 20% of all triple-negative breast cancers may arise from *BRCA1*-methylated subclones of normal cells. Low-level, mosaic *BRCA1* methylation is found in newborns at a frequency resembling that in adults and is twice as frequent in newborn and adult females as compared to males. Lack of correlation in *BRCA1* methylation status between newborns and their parents argue against mendelian inheritance.

### Supplementary Information

**Additional file 1. **Contains Supplementary Methods, Supplementary Figures S1–S10 and Supplementary Table S1–S6.

**Additional file 2. **Contains summary data underlying results for cancer patients.

**Additional file 3. **Contains summary data underlying results for newborns and their parents.

## Data Availability

Raw, processed, and summary data underlying this manuscript have been deposited at NCBI Gene Expression Omnibus under accession number GSE243966 [62]. Summary data is also made available as Additional File 2 and Additional File 3. Haukeland University Hospital and the University of Bergen support the dissemination of research data that has been generated and increased cooperation between investigators. Trial data is collected, stored, and disseminated according to institutional guidelines and in accordance with national laws and regulations to ensure the quality, integrity, and use of clinical data. Signed informed consent forms are stored at each participating hospital and are available for monitoring by regulatory authorities. After publication and upon formal request, raw data, including de-identified individual participant data and a data dictionary defining each field in the data set, will be shared according to institutional procedures. Requests are via a standard pro forma describing the nature of the proposed research and extent of data requirements. Data recipients are required to enter a formal data sharing agreement which describes the conditions for release and requirements for data transfer, storage, archiving, publication, and intellectual property. Requests are reviewed by the EPITAX, DDP, and PETREMAC study teams in terms of scientific merit and ethical considerations, including patient consent. Samples from the MoBa study were analyzed blinded to the identity of the participants. Since *BRCA1* WBC methylation has been found associated with an elevated risk of HGSOC and TNBC [26], under Norwegian law, this means that WBC *BRCA1* methylation status may be considered predictive testing. Thus, our Regional Ethics Committee approved this part of the study provided no identification key or link to clinical information was stored. Thus, all molecular data from newborns in the present study were handled anonymously after analysis. Additional data are available from the authors upon reasonable individual request, but any link to parameters in the MoBa databank is not possible.
